# Comprehensive Thermal Characteristics of Different Cultivars of Flaxseed Oil (*Linum usittatissimum* L.)

**DOI:** 10.3390/molecules26071958

**Published:** 2021-03-31

**Authors:** Jolanta Tomaszewska-Gras, Mahbuba Islam, Liliana Grzeca, Anna Kaczmarek, Emilia Fornal

**Affiliations:** 1 Department of Food Quality and Safety Management, Poznań University of Life Sciences, ul. Wojska Polskiego 31/33, 60-637 Poznań, Poland; mahbuba.islam@up.poznan.pl (M.I.); liliana.grzeca@up.poznan.pl (L.G.); anna.kaczmarek@up.poznan.pl (A.K.); 2 Department of Pathophysiology, Faculty of Medicine, Medical University of Lublin, ul. Jaczewskiego 8b, 20-090 Lublin, Poland; emilia.fornal@umlub.pl

**Keywords:** flaxseed oil, melting, crystallization, oxidative stability, differential scanning calorimetry (DSC)

## Abstract

The aim of this study was to describe the thermal properties of selected cultivars of flaxseed oil by the use of the differential scanning calorimetry (DSC) technique. The crystallization and melting profiles were analyzed depending on different scanning rates (1, 2, 5 °C/min) as well as oxidative induction time (OIT) isothermally at 120 °C and 140 °C, and oxidation onset temperatures (Ton) at 2 and 5 °C/min were measured. The crystallization was manifested as a single peak, differing for a cooling rate of 1 and 2 °C/min. The melting curves were more complex with differences among the cultivars for a heating rate of 1 and 2 °C/min, while for 5 °C/min, the profiles did not differ, which could be utilized in analytics for profiling in order to assess the authenticity of the flaxseed oil. Moreover, it was observed that flaxseed oil was highly susceptible to thermal oxidation, and its stability decreased with increasing temperature and decreasing heating rate. Significant negative linear correlations were found between unsaturated fatty acid content (C18:2, C18:3 n-3) and DSC parameters (OIT, Ton). Principal component analysis (PCA) also established a strong correlation between total oxidation value (TOTOX), peroxide value (PV) and all DSC parameters of thermo-oxidative stability.

## 1. Introduction

Flax (*Linum usitatissimum* L.) is an important plant known and cultivated all over the world mainly for oil and fiber. Originating from West Asia and the Mediterranean region, flaxseed (*Linum usitatissimum*, Latin; meaning “very useful”) has long been cultivated as one of the oldest multi-purpose crops in history [1]. According to the latest report in 2017 from the Food and Agriculture Organization (FAO), the approximate annual flaxseed production worldwide reached about 2.8 million tons [2]. The cultivation of flaxseed and the quality of the oil yield are significantly influenced by factors such as the temperate climate zone, cultivation methods, genotype and biotic and abiotic stresses, seed moisture at harvest, agronomic treatment, etc. [3,4,5,6,7]. The use of flaxseed for human consumption dates back to ancient times, hence the revelation of the flax genome sequence in 2012 has added a new value and attention to the study [3]. The potential nutritional benefits of flaxseed oil are associated with its active biological compounds. Flaxseed oil comprises outstanding quantities of polyunsaturated fatty acids (PUFA), phytoestrogenic lignans (secoisolariciresinol diglucoside, SDG) [8], and an array of antioxidants such as phenolic acids and flavonoids. The beneficial PUFA of flax lipids are α-linolenic acid (ALA), C18:3, n-3 (30–70% of the total fatty acid content), linoleic, C18:2, n-6; (20% of the total fatty acid content), and oleic acid 18:1 (30% of the total fatty acid content) [3]. ALA in flaxseed can be metabolized to eicosapentaenoic acid (EPA) and docosahexaenoic acid (DHA) [9]. Nutrients present in flaxseed oil can play a pivotal role against various inflammatory autoimmune disorders, hypertension, diabetes, menopausal symptoms, and osteoporosis, and improve the condition of the nervous system and proper blood circulation [2,5,10]. Considering the compelling medicinal values, flaxseed and cold-pressed flaxseed oil have been introduced into the 9th edition of European Pharmacopoeia [11]. On the other hand, the presence of higher ALA content in flaxseed oil can make it distinctly susceptible to oxidation [12]. This oil is very sensitive to heat, light, exposure to oxygen and storage time, thus it can rapidly become rancid due to its higher concentration of ALA [11]. 

Different cultivars of flaxseed oil have been examined in various aspects. A low-linolenic variety, belonging to the Solin-type group (Solal), and a traditional linseed one rich in linolenic acid (Bethune) were compared in order to assess their agronomical and qualitative characteristics [11]. The yield, oil content and fatty acid profile of seven flax cultivars from Argentina were also tested [5]. The tocochromanols and fatty acid composition of two groups of flax genotypes were investigated by Trela et al. [3]. The seven cultivars from Russia, Poland, Uruguay, Great Britain and Canada [6] as well as the Szafir variety from Poland [13] and the three types of transgenic flax Linola [14] were examined in terms of the susceptibility of flaxseed oil to peroxidation. 

According to Frega, Mozzon and Lercker [15], the most important parameter for oil quality is determined by its oxidative stability. In addition to the active oxygen method and Rancimat method [12], there are several other analytical methods that have been developed to estimate the freshness or oxidation ratio, i.e., peroxide value (PV), acid value (AV), p-anisidine value (pAV) [16]. However, to minimize the duration of experiments, laborious difficulty and the use of harmful chemicals, instrumental thermoanalytical methods are becoming popular for the characterization of fats and oils. 

Taking into account the limited scientific data on the thermal properties of specified flaxseed cultivars, the aim of this study was to determine the melting and crystallization curves at various scanning rates, as well as thermo-oxidative stability based on isothermal and non-isothermal heating treatment by applying the differential scanning calorimetry (DSC) technique in order to establish comprehensive thermodynamic characteristics for assessing both the authenticity and stability of the flaxseed oil.

## 2. Results

### 2.1. Fatty Acid Composition

Fatty acid composition is substantial for the oxidative stability and physicochemical and nutritional properties, thus it was analyzed in five samples of cold-pressed flaxseed oils, the results of which are presented in Table 1. Three certified cultivars of flaxseed, i.e., Bukoz (FL BU), Dolguniec (FL DL), Szafir (FL SZA, FL SZB) and one sample of an unknown variety (FL NN) were taken for this study. The most abundant fatty acid in all flaxseed oils was α-linolenic fatty acid (ALA, C18:3, n-3), which is the dominant compound and unique feature of this oil. Flaxseed has a very high level of ALA, usually greater than 50% of the total fatty acids. Among the cultivars investigated, ALA varied from 55.91% for sample FL NN to 63.11% for FL SZA. It is notable that for each mean value, differences were significant (*p* < 0.05). The ALA content of five flaxseed oils was quite similar to other studies: in oil from the Szafir seed variety, ALA was found at the level of 65.4% [6]; from the Bukoz seed variety at 58% [3]; between 58.87 and 60.42% was determined in various flaxseed oils from New Zealand [17]; 64% was found in the Bethune variety from Italy [11]; between 50 and 60% in Ethiopian flaxseed cultivars, while in samples of the Canadian flaxseed, ALA was found to range from 52–63% [18], and the highest amount of ALA, which yields 69%, was determined in a recently registered cultivar in Canada (VT 50; Trademark: NuLin) [19]. In turn, a content of ALA below 50% was determined in the seven cultivars from Argentina [5], in five cultivars from Egypt [20], in the oil from fiber-flaxseed of an unknown variety from China (47%) [21] and from Poland [16]. The second most abundant fatty acid in flaxseed oil was oleic fatty acid (C18:1), which yielded between 14.64 and 18.71%, with significant differences between each examined cultivar. The oils FL NN and FL SZB were characterized by the highest percentage of oleic acid (18.46 and 18.71%, respectively). Among the varieties, there were also significant differences in the percentage of linoleic acid (C18:2), for which the highest content (16.56%) was noted for sample FL BU and the lowest (11.14%) for FL SZA. In samples of flaxseed oils, saturated acids were also detected. The highest content of saturated FA (C16:0, C18:0) was found in samples FL NN and FL SZB, and these samples also had the lowest ratio of unsaturated to saturated fatty acids (9.0 and 9.1, respectively), while for FL BU this ratio was the highest, at around 11, which is in agreement with other published results [17]. In order to determine the relations between the content of fatty acids, correlation analysis was carried out, which showed high positive correlations between C18:1 and C18:0 (r = 0.93), between C18:1 and C16:0 (r = 0.85) and a negative correlation between C18:1 and C18:3 (r = −0.59). Similarly, a high positive correlation (r = 0.83) between oleic and palmitic acid was found by other researchers [6], while a negative correlation between oleic and linolenic acid was also confirmed by other studies [3,17,18].

### 2.2. Color Measurement

Color is an important quality determinant, significant for consumers’ acceptability; therefore, in order to determine the parameters describing color, measurements of reflectance at a spectrum of wavelengths from 400 to 740 nm and the calculation of three parameters of L*, a*, b* were performed, with results given in Table 2. Among the five oil samples analyzed, significant differences were found in mean values of L*, a* and b*, and it is noticeable that flaxseed oil samples were characterized by a high value of b*, attributable to yellow color. The highest yellowness (b*), redness (a*) and lightness (L*) were noted for the Bukoz variety FL BU (136.58, 8.99, 87.86, respectively). Similar results were obtained by Choo, Birch and Dufour [17].

### 2.3. DSC Crystallization Profiles of Cold-Pressed Flaxseed Oils

**Figure 1:**
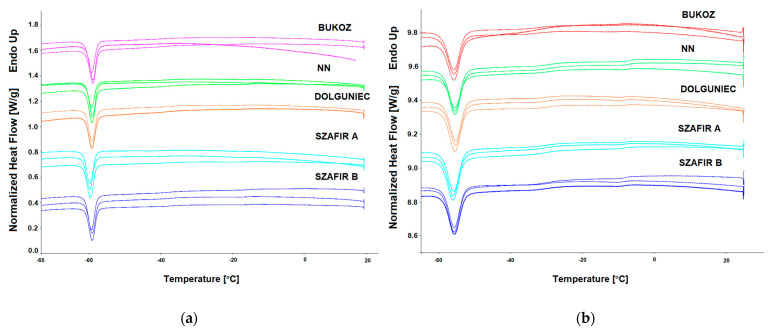
DSC crystallization curves of cold-pressed flaxseed oils from different cultivars obtained with various cooling rates: (**a**) Cooling rate 1 °C/min; (**b**) Cooling rate 2 °C/min.

The thermal properties of fats and oils can be described by measuring thermal behavior during the phase transition, such as crystallization or melting, but also by determining thermal stability during chemical reactions of oxidation. All these measurements were carried out using the analytical technique of differential scanning calorimetry. Since the DSC procedure for investigating phase transition involved first cooling the oil sample before heating, the crystallization curves will be discussed first. In Figure 1, the crystallization profiles of cold pressed flaxseed oils obtained by two different scanning rates are shown, and in Table 3 the results of crystallization temperature and enthalpy are presented. As can be seen on the cooling curves (Figure 1), one crystallization peak for both scanning rates (1 and 2 °C/min) was detected. Due to the high content of unsaturated fatty acids (Table 1), crystallization phase transition takes place for both scanning rates (1 and 2 °C/min) below a temperature of −50 °C, where for the scanning rate 1 °C/min it occurred for all five flaxseed oils being tested in a narrow range from −55.35 to −54.59 °C, while for the scanning rate 2 °C/min the temperature ranged from −60.24 to −59.1 °C. For both scanning rates (1 and 2 °C/min), the mean values of temperatures between cultivars did not differ significantly. However, it was observed that for the cultivar characterized by the highest UFA/SFA ratio (FL BU, ratio = 11, Table 1), the lowest crystallization temperature was observed for both scanning rates (1 and 2 °C/min). Correlation analysis between the ratio of UFA/SFA and crystallization temperature showed that there is a negative linear correlation with Pearson’s correlation coefficient (r) −0.87 for rate 1 °C/min and −0.88 for rate 2 °C/min. The second parameter measured for the crystallization process was the enthalpy of the transition, which was measured as the area of the peak. The mean values of enthalpy did not differ significantly between the various cultivars of flaxseed oil for scanning rate 1 °C/min as well as for rate 2 °C/min. However, the values of enthalpy obtained for scanning rate 2 °C/min were lower than for rate 1 °C/min, for which the mean value calculated from all five cultivars was 34.12 J/g, while for scanning rate 2 °C/min it was −28.23 J/g. The results obtained for scanning rate 1 °C/min correlate well with the study presented by Teh and Birch [22], which reported a peak temperature at the value of −53.79 °C and enthalpy 40.98 J/g. There are no studies performed using a scanning rate of 2 °C/min to compare the results obtained. 

### 2.4. DSC Melting Profiles of Cold-Pressed Flaxseed Oils 

**Figure 2:**
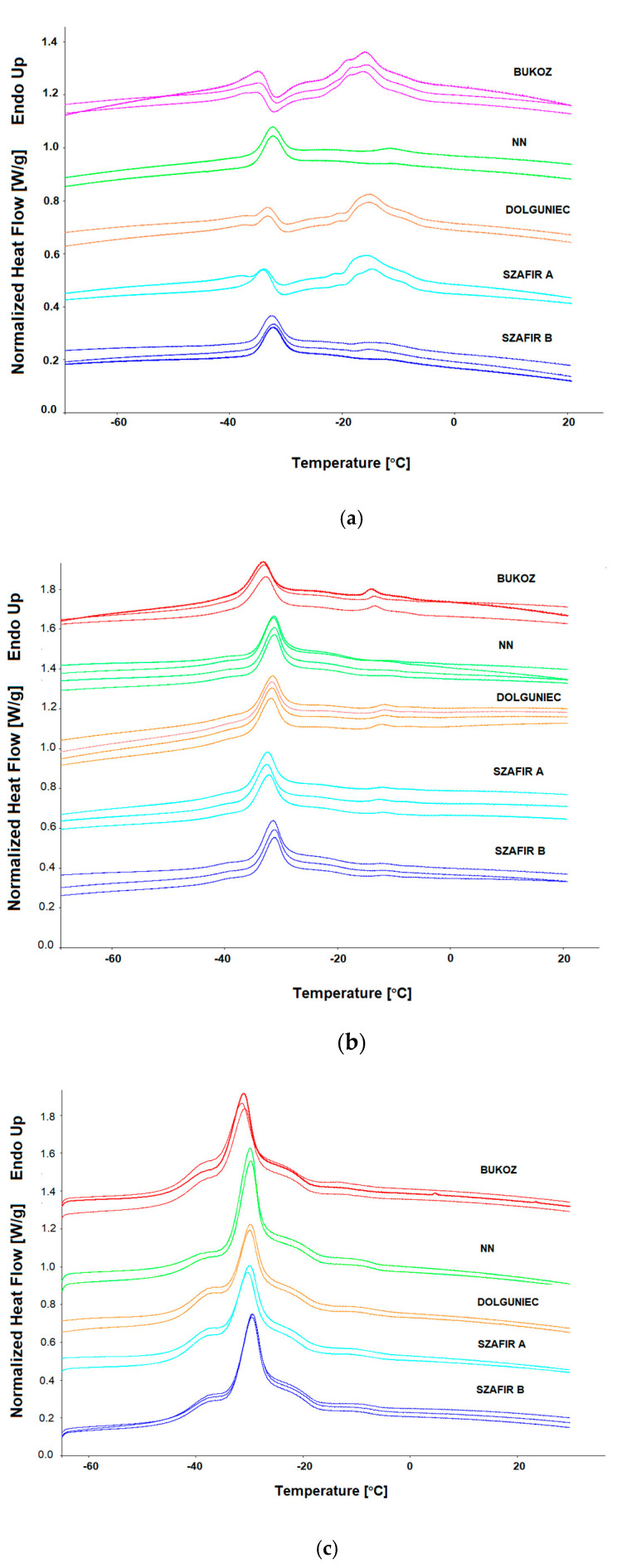
DSC melting curves of cold-pressed flaxseed oils from different cultivars obtained with various heating rates: (**a**) Heating rate 1 °C/min; (**b**) Heating rate 2 °C/min; (**c**) Heating rate 5 °C/min.

Following the crystallization process, the melting phase transition of cold-pressed flaxseed oils was also investigated at different scanning rates. Figure 2a–c depicts melting curves obtained at different heating rates (1, 2, 5 °C/min). As can be seen in Figure 2, the melting profiles of flaxseed oil differ from crystallization curves. Firstly, the most discernible difference between crystallization and melting curves can be observed in the shape of the curves. Curves obtained during crystallization are manifested by only one peak, while melting curves are more complex because there are more peaks not separated. In contrast to the crystallization process, where the shape of the curves was similar for both scanning rates, in the melting process the shape of the curves and the size of the peaks (width and height) are affected by the scanning rate. Figure 2a shows that the melting profiles of five different cultivars of flaxseed differ for scanning rate 1 °C/min. In Table 4, the parameters of the melting peaks (temperatures and enthalpies) obtained by scanning rate 1 °C/min are given. The lowest values of melting temperatures and the highest of total enthalpy (ΔHm total), significantly different from other mean values, were recorded for sample FL BU (Tm1 = −35.69 °C; Tm2 = −13.77 °C, ΔHm total = 127.91 J/g). All the melting curves of the five oil samples analyzed at this rate exhibited one common peak Tm1 in the range of −31.73 to −35.69 °C. The second peak Tm2, appearing within the range of −10.26 to −13.77 °C, differed in size among the cultivars (Figure 2a). Teh and Birch [22] reported similar results of temperature values obtained at rate 1 °C/min, i.e., −36.28 and −15.43 °C for both peaks. 

Comparing the shape of curves obtained by scanning rate 1 °C/min, it can be seen that the melting profiles for flaxseed cultivars of Bukoz, Dolguniec and Szafir A are similar, where two peaks, Tm1 and Tm2, are clearly visible. In the case of NN and Szafir B flaxseed oil samples, the huge reduction in the peak Tm2 at around −10 °C was observed. 

Figure 2b shows the melting curves of five flaxseed oil samples obtained with a scanning rate of 2 °C/min. The shape of the curves differed from curves obtained with a scanning rate of 1 °C/min. The most significant difference compared to the curves with a rate of 1 °C/min is the reduction in the second peak or its complete disappearance, as in the case of the FL NN sample. This indicates that the heating rate of 2 °C/min is too fast for polymorphic transition to occur, as it was by the rate 1 °C/min. Table 5 presents the results of melting transition with a scanning rate of 2 °C/min. There are no significant differences between the five samples of oils in mean values of peak temperatures, although the values of melting enthalpy are significantly different. The highest total enthalpy was observed for sample FL SZA (50.84 J/g) and lowest for FL NN (43.12 J/g). Similarly, as for rate 1 °C/min, the lowest values of melting temperatures were noted for the Bukoz variety (FL BU), Tm1 = −32.89 °C and Tm2 = −11.08 °C, and the highest for sample FL NN, Tm1 = −30.34 °C, and a second peak was not detected.

Figure 2c depicts the melting curves of five oil samples obtained with a heating rate of 5 °C/min. It can be seen that all the curves differ from the curves obtained by a scanning rate of 1 and 2 °C/min. However, they do not differ in shape among the cultivars. All curves are very similar and repeatable with the main peak at a temperature between −29.45 and −31.88 °C, as is shown in Table 6. There were no significant differences in the mean values of temperature nor in enthalpy. However, the lowest values of melting temperatures were obtained for FL BU and the highest values for sample FL NN, as in the case of a heating rate of 1 and 2 °C/min. Moreover, it can be seen from all curves that the main peak is composed of more than one peak, and two pronounced shoulders on both sides of this peak are visible. The first shoulder is from around −47 °C to −35 °C, and the second is from −27 °C to −17 °C. Additionally, a small separate shoulder was identified at around—13 °C. The results are consistent with those obtained by Zhang et al. [10], who studied the effect of heating on the DSC melting curve of flaxseed oil. In their experiment, they similarly mentioned about one major peak at −31 °C and two endothermic shoulders with a maxima at −38 °C and at −24 °C. An endothermic event was also recognized at −13 °C. Our results obtained for scanning rate 5 °C/min also correlated well with the study of Zhang et al. [21], which reported a peak temperature at the value of—32.53 °C and −30.65 °C and enthalpies of 62.15 J/g and 57.85 J/g for two different flaxseed oils. 

Considering the impact of the fatty acid composition of flaxseed oils on the melting temperatures, Pearson’s correlation analysis was performed between temperature value Tm1 and ratio UFA/SFA for each scanning rate. The analysis revealed that for all heating rates, Pearson’s correlation coefficients were statistically significant (*p* < 0.05), and the values r were −0.98, −0.97, and −0.99, respectively, for scanning rates 1, 2, and 5 °C/min.

### 2.5. Isothermal Determination of Oxidative Stability by DSC Non-Isothermal Determination of Oxidative Stability by DSC

**Figure 3:**
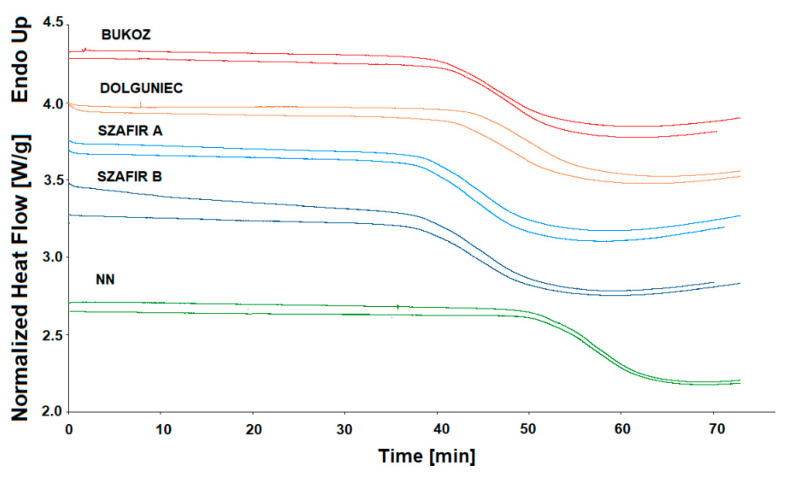
DSC isothermal oxidation curves obtained at 120 °C for cold-pressed flaxseed oils.

**Figure 4:**
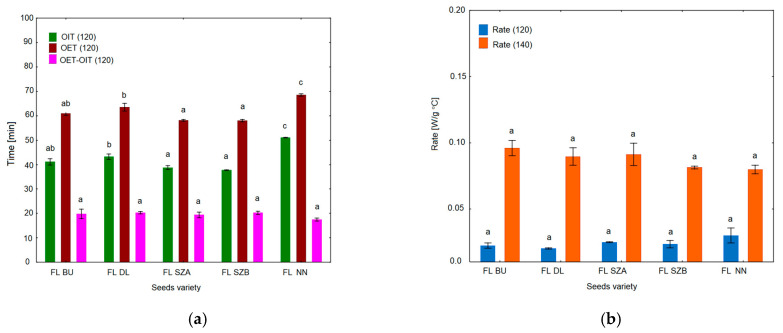
Oxidative stability parameters of five cold-pressed flaxseed oils determined isothermally by DSC at 120 °C: (**a**) Oxidation induction time OIT (min), oxidation end time OET (min), the oxidation duration Δt (min). (**b**) Rate of oxidation at 120 and 140 °C. Different letters (a, b, c) indicate significant differences (*p* < 0.05). FL BU (Bukoz Flaxseed cultivar), FL DL (Dolguniec Flaxseed cultivar), FL SZA, FL SZB (Szafir Flaxseed cultivar), FL NN (Unknown Flaxseed cultivar).

Oxidative stability is one of the most important quality features of all edible fats and oils because lipid peroxidation leads to their nutritional and sensory deterioration. Polyunsaturated fatty acids, the lipid compounds most prone to oxidation in the presence of light, oxygen or high temperature, can form free radicals, which are transformed into aldehydes and ketones responsible for undesirable flavor. Using the DSC technique, it is possible to create favorable conditions for lipid peroxidation, i.e., increased temperature and oxygen atmosphere. The DSC method provides information regarding oils’ resistance to thermal oxidation, which can be measured isothermally and non-isothermally. Figure 3 depicts the DSC curves of the isothermal determination of oxidative stability at 120 °C of the five cold-pressed flaxseed oils. All DSC curves show a sharp exothermic decline at the initiation of the oxidation process due to heat evolving during the oxidation reaction. Only in the case of sample FL NN compared to other varieties was the point of the curve’s decline shifted to a higher value of time. Figure 4 shows graphs with results calculated from oxidation curves presented in Figure 3. Various parameters of oxidative stability were analyzed, i.e., oxidation induction time (OIT), oxidation end time (OET), the length of the oxidation process Δt (OET-OIT) and the rate of oxidation. The time required for the decline in exotherm was taken as the induction time OIT (min). As can be seen in Figure 3 and Figure 4, the best oxidative stability was exhibited by oil sample FL NN, for which oxidation started at 51 min, while significantly lower OIT values were registered for the sample FL DL at 43 min and FL BU at 41 min. The Szafir variety (FL SZA, FL SZB) was characterized by the worst oxidative stability, with OIT values around 37–38 min, which was significantly different from the rest of the oil samples. Considering the second parameter OET, determined from the oxidation curve at 120 °C, it was noted that this parameter followed in the same order as was observed for OIT, and the mean values differed significantly between each other in a similar way as for OIT. On the other hand, another factor Δt, which measures the oxidation duration, ranged from 17 to 20 min with no significant differences among the cultivars. The last parameter calculated from the DSC oxidation curve at 120 °C was the rate of oxidation, which can express the speed of the oxidation process. Among the various cultivars, the mean values did not differ significantly, and ranged between 0.02 and 0.03. 

In summary, five different samples of flaxseed oils started to be oxidized isothermally at 120 °C between 37 and 51 min, and oxidation finished between 58 and 68.5 min. The sample FL NN was characterized by the highest and significantly different values of OIT and OET. However, the duration and rate of oxidation at 120 °C did not differ significantly among the varieties. 

**Figure 5:**
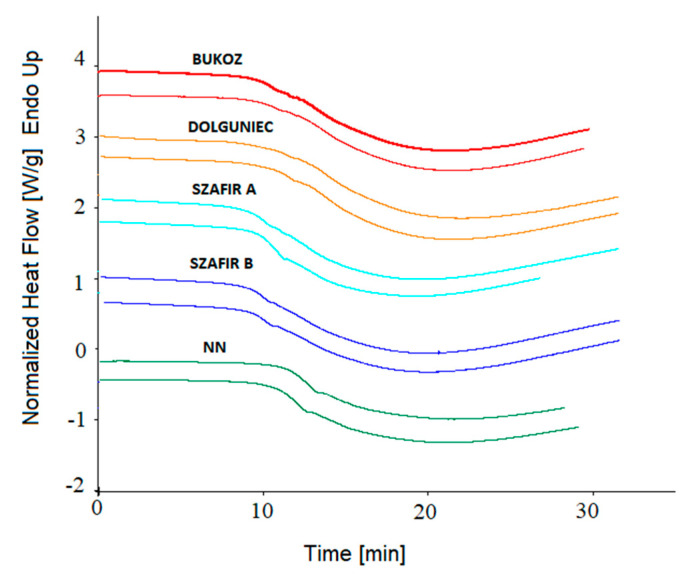
DSC isothermal oxidation curves obtained at 140 °C for cold-pressed flaxseed oils.

**Figure 6:**
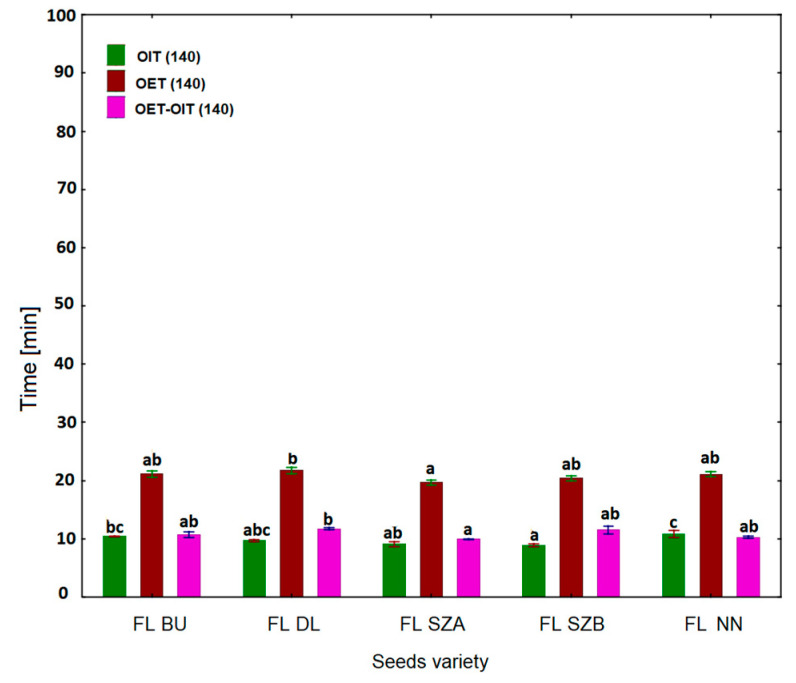
Oxidative stability parameters of five cold-pressed flaxseed oils determined isothermally by DSC at 140 °C: Oxidation induction time OIT (min), oxidation end time OET (min), the oxidation duration Δt (min). Different letters (a, b, c) indicate significant differences (*p* < 0.05). FL BU (Bukoz Flaxseed cultivar), FL DL (Dolguniec Flaxseed cultivar), FL SZA, FL SZB (Szafir Flaxseed cultivar), FL NN (Unknown Flaxseed cultivar).

To confirm the observed resistance of some flaxseed varieties to oxidation, a further measurement was carried out by isothermal DSC at higher temperatures than 120 °C, i.e., 140 °C. The curves are shown in Figure 5 and the results are presented in Figure 6. It was observed that the initiation point of oxidation appearing as the decline of exotherm (OIT) occurred much sooner in the case of 140 °C than at 120 °C. The mean values for 140 °C were five times lower than for 120 °C, and ranged between 8 and 11 min. It is also worth noting that the highest OIT values were observed for the FL NN sample and the lowest for the Szafir samples (FL SZA, FL SZB), as for the 120 °C. The values of the OET parameter were in a similar order as for OET at 120 °C and as for OIT at 120 °C and 140 °C; the highest values were noted for FL NN and FL DL (21.82 and 21.93 min, respectively), and the lowest for Szafir A and B (19.51 and 20.17 min, respectively). Subsequently, the results for the length of oxidation (Δt) at 140 °C were analyzed (Figure 6). It was observed that the Szafir variety (FL SZA) was completely oxidized in the shortest time, around 10 min, and this value was significantly different from the values obtained for the remaining four oil samples (*p* < 0.05). Figure 4b shows the results of calculating the oxidation rate for 140 °C. Compared to the results of the rate obtained at 120 °C, it can be noted that for 140 °C the oxidation rate was three times higher for all varieties. Samples FL SZA and FL BU were oxidized with the highest rate (0.09), whereas the other three varieties showed a rate of 0.08. However, as was the case at 120 °C, there were also no significant differences among cultivars (*p* > 0.05).

By way of conclusion, it can be stated that for the DSC isothermal measurement of the oxidation stability of flaxseed oils, both measurements at 120 and 140 °C confirmed the best resistance to oxidation of sample FL NN and the worst of the Szafir variety (FL SZA, FL SZB). 

### 2.6. Non-Isothermal Determination of Oxidative Stability by DSC 

**Figure 7:**
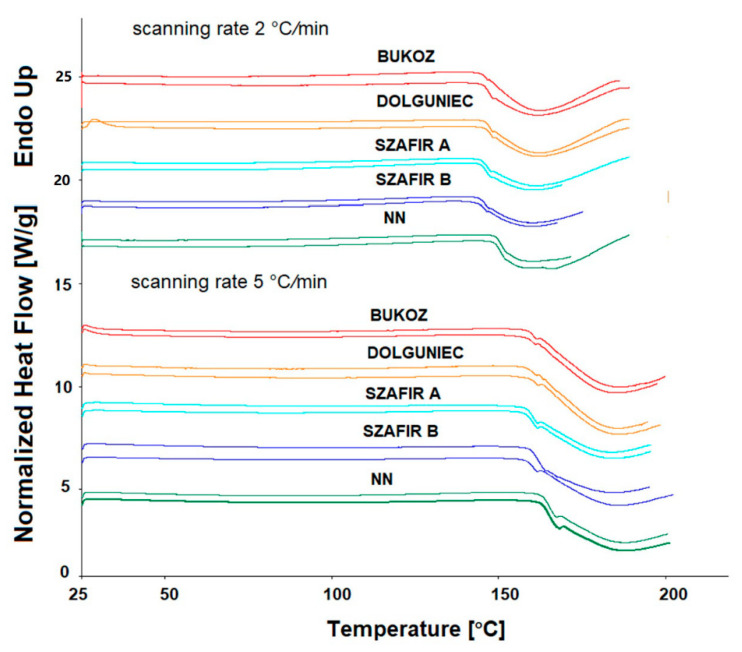
DSC non-isothermal oxidation curves obtained at a scanning rate 2 and 5 °C/min for cold-pressed flaxseed oils.

**Figure 8:**
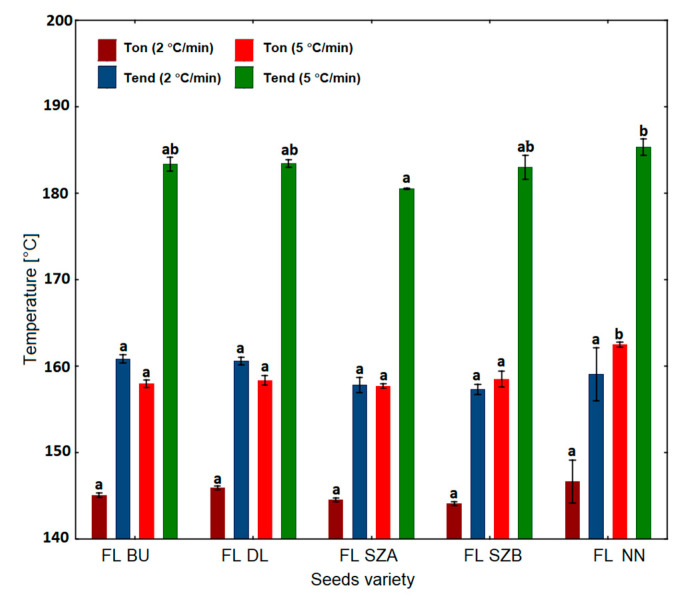
Oxidative stability parameters of five cold-pressed flaxseed oils determined non-isothermally by DSC at a scanning rate of 2 and 5 °C/min. Different letters (a, b) indicate significant differences (*p* < 0.05). FL BU (Bukoz Flaxseed cultivar), FL DL (Dolguniec Flaxseed cultivar), FL SZA, FL SZB (Szafir Flaxseed cultivar), FL NN (Unknown Flaxseed cultivar).

Figure 7 and Figure 8 demonstrate the results of the non-isothermal measurements of thermal stability by DSC of the five samples of flaxseed oils carried out with a scanning rate of 2 and 5 °C/min, for which the values of onset temperature (Ton) and end temperature (Tend) were determined. Considering the scanning rate 2 °C/min, it can be observed that for the Szafir variety (FL SZA, FL SZB), the oxidation initiation temperature (Ton) was the lowest, i.e., 144.5 and 144.1 °C, respectively, while for sample FL NN, the highest value was noted at 146.6 °C. However, all mean values of Ton were not significantly different among the five flaxseed oil varieties. Along with the determination of Ton, Tend values were also collected, which express the end of the oxidation process. As in the case of Ton, for the Szafir variety (FL SZA, FL SZB) the values of Tend were the lowest, i.e., 157.8 and 157.3 °C, respectively, while the highest values were observed for FL BU and FL NN (160.8 and 160.6 °C). However, there were no significant differences between the mean values of Ton and Tend among the five oils at a scanning rate of 2 °C/min. The oxidative behavior of oils under increasing temperature was further tested for a higher heating rate, i.e., 5 °C/min (Figure 7 and Figure 8). The determination of onset time (Ton) for rate 5 °C/min confirmed the best stability of the sample of FL NN, reaching the highest level and significantly different from the remaining four oils at a value of 162.50 °C (*p* < 0.05). The Ton values for the remaining oils ranged between 157.7 and 158.5 °C and they do not differ significantly. Similarly, the parameter of the end of oxidation (Tend) was highest and significantly different for FL NN. Summing up, the non-isothermal DSC technique showed that the Szafir seed variety was oxidized at the lowest temperatures for both 2 and 5 °C/min, along with the highest values for the sample FL NN variety, indicating significantly higher resistance to oxidation than for other varieties in both cases of rate. 

### 2.7. Peroxide, p-Anisidine, TOTOX and Acid Value

**Figure 9:**
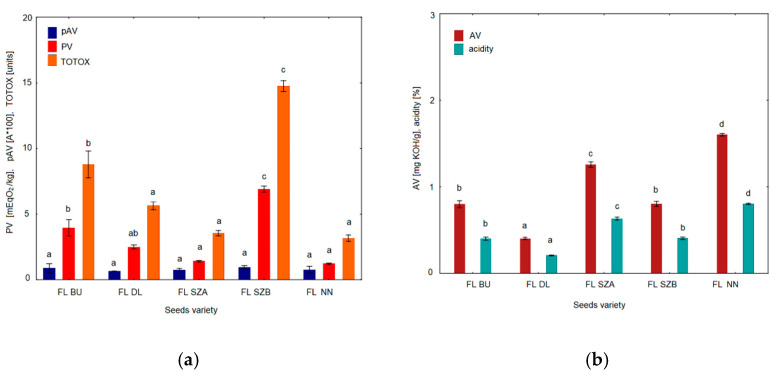
Oxidative stability parameters of five cold-pressed flaxseed oils: (**a**) Peroxide value (PV), p-anisidine value (pAV), total oxidation rate (TOTOX) value; (**b**) Acid value (AV), acidity. Different superscript letters (a, b, c, d) indicate significant differences (*p* < 0.05). FL BU (Bukoz Flaxseed cultivar), FL DL (Dolguniec Flaxseed cultivar), FL SZA, FL SZB (Szafir Flaxseed cultivar), FL NN (Unknown Flaxseed cultivar).

In order to compare the results of the oxidative stability determined by DSC with traditional chemical determinations, various analyses were carried out, i.e., peroxide value (PV), as a measure of primary oxidation products, p-anisidine value (pAV), as an indicator of nonvolatile secondary oxidation products, and acid value (AV) to measure the degree of hydrolytic changes. Moreover, the parameter of total oxidation value (TOTOX) was calculated based on the results of p-anisidine value and peroxide value (TOTOX = pAV+2 PV). Figure 9a depicts the values obtained from chemical analysis of pAV and PV, as well as the calculated parameter TOTOX. Since all the peroxide values obtained were below 7 meqO_2_/kg, p-anisidine values were lower than 1.0 and TOTOX below 15, it can be assumed that all the oils studied were of good quality because the requirements of the Codex Alimentarius standard [23] for a peroxide value (<15 meqO_2_/kg) and of pAV <2.0 were met. Among all the cultivars, the highest values of all three parameters were noted for the sample FL SZB: pV = 6.9 mEqO_2_/kg, pAV = 0.94, and TOTOX = 14.74. In turn, the lowest value of pV was 1.21 mEqO_2_/kg and TOTOX was 3.17 for sample FL NN, although for the sample FL DL, the lowest value of pAV = 0.65 was measured. Only in the case of pAV did the mean values not differ significantly among the cultivars (*p* < 0.05). Figure 9b shows the results of acid value and acidity measurement. The acid value is the measure of free fatty acids as a result of the hydrolytic breakdown of triglyceride molecules. The results show that acid values ranged between 1.6 and 0.4 mg KOH/g. This confirmed that the flaxseed oil samples represent good quality, as their acid values did not exceed the maximum limit of 4.0 mg KOH/g of oil according to the Codex Alimentarius standard [23]. Similar results of peroxide, anisidine and TOTOX values were obtained for seven cold-pressed flaxseed oils sold in New Zealand by Choo, Birch and Dufour [17].

## 3. Discussion

In this study, differential scanning calorimetry was used to characterize the thermal behavior of various cultivars of flaxseed oil in terms of melting and crystallization profiles as well as thermo-oxidative stability. The crystallization analysis showed that there were no significant differences between cultivars in peak temperature and enthalpy, although for oil with the highest UFA/SFA ratio, the lowest crystallization temperature was observed for both scanning rates (1 and 2 °C/min). Correlation analysis between the ratio of UFA/SFA and crystallization temperature showed a significant, negative linear correlation with coefficients r = −0.87 for rate 1 °C/min and r = −0.88 for rate 2 °C/min. This observation is in line with other studies carried out on various edible oils [24].

The effect of cooling rate on the crystallization peak temperatures and enthalpies was also found. The results obtained from crystallization indicated that the higher the cooling rate, the lower the peak temperatures and the lower the enthalpy of transition. This statement is in agreement with previous research conducted on butterfat [25] and other vegetable oils [26]. 

The melting profiles were also analyzed by using various heating rates. Despite variation in the shape of the melting profile between the various scanning rates, one common peak was found at around −30 °C (Tm1) for all heating rates. Interestingly, for heating rate 1 °C/min, differences in the shape of the melting curve were also observed between cultivars. For the cultivar of Bukoz, Dolguniec and Szafir A, two peaks were identified, while for NN and Szafir B the second peak was reduced. This can indicate that the first peak, appearing at lower temperatures (Tm1), is the result of melting a less stable α polymorph that was formed during cooling, and the second peak (Tm2) could arise from polymorphic transition. The lower enthalpy for peak Tm1 than for peak Tm2 in the case of samples FL BU, FL DL, and FL SZA can confirm this statement because the higher the enthalpy, the more stable the polymorph. The highest rate of polymorphic transition was observed for the cultivar Bukoz (FL BU), with an enthalpy (ΔHm Peak 2) noted for the peak Tm2 of 92.80 J/g and the lowest for the Szafir (FL SZB) of 8.09 J/g (Table 4). The lower enthalpy for the second peak was also observed for sample FL NN. The similar behavior of flaxseed oil with recrystallization and polymorphic transitions of metastable forms at a heating rate of 1 °C/min was noted by Teh and Birch [22]. Oomah and Sitter [27] also observed two similar transitions, the first between −35 and −33 °C and the second between −25 and −24 °C indicative of crystalline melting corresponding to the α and β polymorphic forms. Similarly, a minor transition at −14 °C was detected.

Analyzing these results, the question may arise as to what kind of compositional differences affect this different polymorphic behavior, particularly in the case of the sample FL SZB and FL NN. As the results of composition indicate (Table 1), for these two samples of oils (FL SZB and FL NN) the highest percentage of oleic fatty acid (18:1), i.e., 18.46 and 18.71%, respectively, and the lowest ratio of unsaturated to saturated fatty acids (9.1 and 9.0, respectively) were observed. In turn, for these samples the total saturated fatty acid level was highest (9.88 and 10.01%, respectively). This indicates that the content of saturated and unsaturated fatty acid is crucial for flaxseed triacylglycerols’ polymorphic behavior, as well as for the peak melting temperatures. For samples with the highest percentage of saturated fatty acid (FL SZB and FL NN), the highest melting temperatures were observed (−31.43 and −31.73 °C, respectively, for Tm1, and −10.87 and −10.26 °C, respectively, for Tm 2). Regarding the influence of the composition of fatty acids on the melting temperatures, correlations analysis revealed that there was a strong negative linear correlation between melting temperature value (Tm1) and ratio UFA/SFA for all scanning rates. Statistically significant (*p* < 0.05) Pearson’s correlation coefficients were obtained, i.e., −0.98, −0.97 and −0.99, respectively, for scanning rates 1, 2, and 5 °C/min. This observation was previously noted by Tan and Che Man [24]. 

Based on these results, it can be assumed that the heating rate 1 °C/min can be considered for the differentiation of flaxseed oil cultivars, while the heating rate 5 °C/min can be utilized as a fingerprint for the authenticity assessment of flaxseed oil.

The DSC technique is also a very convenient method for measuring thermo-oxidative resistance to oxidation caused by oxygen and elevated temperature. It can be measured isothermally at various temperatures or non-isothermally, in dynamic mode with various heating rates. Five different samples of flaxseed oils started to be oxidized isothermally at 120 °C between 37 and 51 min, and oxidation finished between 58 and 68.5 min, while at 140 °C the OIT values were around 4–5 times lower. Flaxseed oil sample FL NN exhibited significantly higher values of OIT and OET than the rest of the oils. The worst resistance to oxidation was noted for the Szafir variety (FL SZA, FL SZB). The non-isothermal DSC technique confirmed these observations. The Szafir seed variety oxidized at the lowest temperatures for both rates 2 and 5 °C/min (144 °C and 157 °C, respectively), along with the highest values for the sample FL NN variety (146.6 °C and 162.5 °C, respectively), thus confirming significantly higher resistance to oxidation than for other varieties in both cases of the rate. The duration and rate of isothermal oxidation analysis at 120 and 140 °C did not differ significantly among the cultivars. However, for 140 °C the rate was three times higher than for 120 °C. In another experiment with five flaxseed oils of the Szafir cultivar, pressure differential scanning calorimetry (PDSC) isothermally at various temperatures and with the oxygen flow rate of 100 mL/min was carried out by Symoniuk, Ratusz and Krygier [13]. They obtained OIT values at 120 °C between 21.20 and 24.34 min and at 140 °C between 4.33 and 4.97 min.

**Figure 10:**
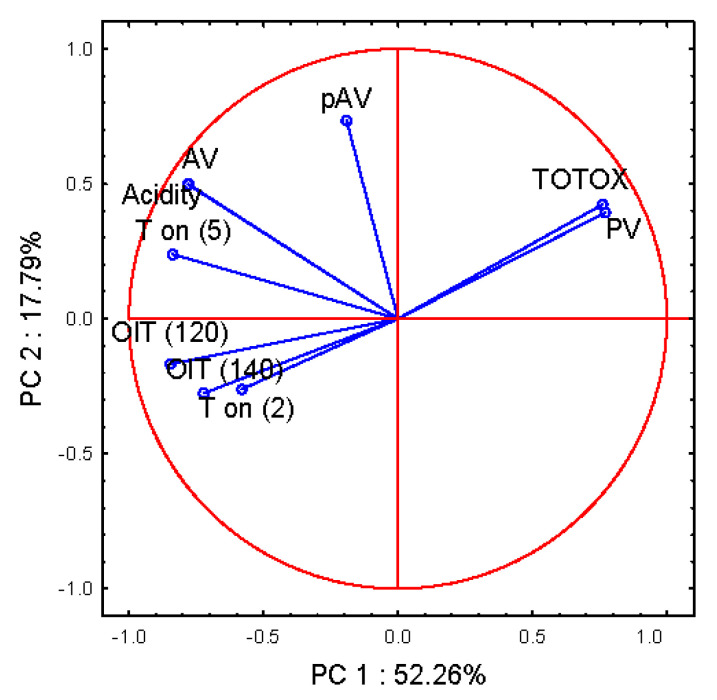
Oxidative stability parameters of five cold-pressed flaxseed oils.

In order to explain the variation in thermal resistance to oxidation among the cultivars, correlation analysis between fatty acid composition and DSC isothermal and non-isothermal results was performed, the results of which are presented in Table 7. As can be seen, high Pearson’s correlation coefficients were obtained between fatty acid C18:3 and all DSC parameters, except for the parameter of non-isothermally measured onset temperature (Ton) at the scanning rate of 2 °C/min. It can be concluded that α-linolenic acid is predominantly responsible for the thermal oxidation of flaxseed oil. This statement is in agreement with the findings of other researchers [28]. In order to establish the relations between thermo-oxidative stability and stability determined by chemical methods, principal component analysis was performed. This is a method for detecting structure in the relationships between variables and to classify the objects [29]. Principal component analysis (PCA) was used to describe the oxidative stability of flaxseed oil measured by different methods. Various variables were used for PCA: from isothermal DSC oxidation determination at 120 and 140 °C: OIT(120), OIT(140) and non-isothermal onset temperature at a heating rate of 2 and 5 °C/min: Ton (2), Ton (5), as well as from the chemical determination of oxidation stability: PV, pAV, AV, acidity, TOTOX. Using the graphical criterion, the first six principal components, which explain 99.85% of the total variance, were derived. Figure 10 depicts the plot of loadings, which visualizes relations between variables by analyzing the first two principal components: PC1 and PC2. The horizontal axis corresponds to PC1, and the vertical to PC2. The closer the variable is to the circle, the more it is correlated with the component. The graph obtained shows that the first two PCs describe 70.05% of the initial variability, where the first PC1 explains the observed variability in 52.26% and PC2 in 17.79%. The first component (PC1) describes the thermo-oxidative stability measured by parameters such as OIT (120), OIT (140) and Ton (2), Ton (5), as well as PV and TOTOX. The first PC is positively correlated with PV and TOTOX, and negatively correlated with the rest of the variables, which were taken for the analysis, except pAV. From all sets of DSC variables which are related to PC1, with all being located on the negative side (negative correlations with PC1), the strongest factor loadings were noted for OIT (120), being −0.85, and for Ton (5) of −0.84. On the opposite side of PC1, the variables of PV and TOTOX are located, which means that there are negative correlations between them and DSC parameters. This observation confirms that the greater the peroxide value, the lower the DSC parameters, i.e., the lower the thermo-oxidative stability. However, it can also be seen that variables of PV and TOTOX are strongly related to PC1 (0.69 and 0.68, respectively), as well as to PC2 (0.56 and 0.58, respectively). Figure 10 also shows no correlation of DSC parameters with anisidine value (pAV), as they are located perpendicular to each other, thus the pAV variable is more related to PC2 with a factor loading of 0.73.

## 4. Materials and Methods

### 4.1. Materials

We obtained three certified cultivars of flaxseed, i.e., Bukoz (FL BU), Dolguniec (FL DL), Szafir, from two different suppliers (FL SZA, FL SZB) and one sample of unknown variety (FL NN). All were brown-seeded flaxseed varieties, collected in 2019 from different parts of Poland, and were used for oil cold-pressing at a temperature under 50 °C. The pressed oils were left for 24 h for decantation and kept in brown glass bottles at freezing temperature (−80 °C).

### 4.2. Fatty Acid Composition

The percentage fatty acids composition was determined by GC-FID. Fatty acid methyl esters were prepared according to the AOCS Official Method Ce 2-66 [30]. Two drops of fat were dissolved in 1 mL of hexane (for HPLC, Sigma Aldrich, Sp. z o.o. Poznan, Poland). A total of 1 mL of 0.4 N sodium methoxide was added. Samples were stirred and left for 15 min, then 5 mL of distilled water was added after and the top layer was taken. Fatty acid methyl esters were analyzed using a Trace 1300 chromatograph (Thermo Fisher Scientific, Waltham, Massachusetts, USA). Separation was performed on a Supelcowax 10 capillary column (30 m × 0.2 mm × 0.2 μm). The injection was performed in splitless mode. The sample volume was 1 µL and hydrogen was used as the carrier gas. The initial furnace temperature was 160 °C, and was increased from 12 °C/min to 220 °C. A temperature of 220 °C was maintained for 20 min. Fatty acid methyl esters were identified on the basis of comparing the retention times in the sample and in the 37-Component FAME Mix (Supelco). 

### 4.3. Color Measurement

Color measurements of oils were carried out according to [17], using the Konica Minolta CM-5 spectrophotometer (Konica Minolta, Inc., Tokyo, Japan) and SpectraMagicNx software (Konica Minolta, Inc., Tokyo, Japan). The instrument was calibrated prior to starting the analysis transmission chamber that can accurately measure both translucent and transparent liquid samples using a CM-A213 zero calibration plate (black calibration), followed by distilled water in a 10 mm CM-A98 glass cuvette (white calibration). The research was conducted using the Hunter Lab scale. Reference standard L*a*b* values were pre-defined and oil color was measured within the defined range. Parameter L* was in the range of 0–100, and denoted the brightness of the color from black to white. Similarly, the a* parameter, depending on the range, determined green (below 0) and red (above 0) tinge. Another parameter determined was the b* parameter, which in the case of negative values defined the color blue, but in the case of positive values—yellow. The samples used were analyzed in three replications.

### 4.4. DSC Melting and Crystallization Analysis

Melting and crystallization analysis of flaxseed oils was carried out with modifications according to the method used for butterfat [31]. A Perkin Elmer differential scanning calorimeter DSC 8500 PerkinElmer (Waltham, Massachusetts, USA), equipped with an Intracooler II and running under Pyris software (Perkin Elmer, Waltham, Massachusetts, USA), was used to examine the melting and crystallization properties of the flaxseed oil. Nitrogen (99.999% purity) was the purge gas. The DSC calorimeter was calibrated using indium (m.p. 156.6 °C, ∆H_f_ = 28.45 J/g) and n-dodecane (m.p. −9.65 °C, ∆H_f_ = 216.73 J/g). Samples of ca. 6–7 mg were weighed into aluminum pans of 20 μL (Perkin Elmer, No. 0219-0062, Waltham, Massachusetts, USA) and hermetically sealed. The reference was an empty, hermetically sealed aluminum pan. Prior to analysis, the samples were heated at 30 °C for 5 min to melt all crystals and nuclei. The samples were cooled at scanning rate 1 and 2 °C/min and heated at scanning rates 1, 2, 5 °C/min. For each measurement at a given scanning rate, the calibration procedure was completed with the correct scanning rate. Crystallization curves were recorded from 30 to −65 °C, then, following cooling, melting curves were obtained from −65 to 30 °C. The temperature of each peak (Tp), the enthalpy of melting or crystallization ∆H [J/g] were measured from cooling or heating curves. All measurements were performed in triplicate for each sample.

### 4.5. Determination of Oxidative Stability by DSC

Oxidative stability was determined by following the ISO standard [32], and also implementing the ASTM procedure [33]. Oil samples were studied in a DSC 7 Perkin Elmer (Norwalk, Connecticut, USA) along with an Intracooler II operated with Pyris software (Perkin Elmer, Waltham, Massachusetts, USA). Both the isothermal and non-isothermal protocols were followed to determine the oxidative stability characteristics of the oils. The instrument was calibrated using indium (m.p. 156.6 °C, ΔH_f_ = 28.45 J/g) and n-dodecane (m.p. −9.65 °C, ΔH_f_ = 216.73 J/g), while 99.99% pure nitrogen gas was used as the purge gas. For the isothermal program, temperatures of 120 °C and 140 °C were maintained with a constant oxygen flow of 20 mL/min. For the non-isothermal program, curves were obtained by operating a scanning rate of 2 °C/min and 5 °C/min, respectively. Based on the resulting curves, parameters denoted as oxidation induction time (OIT), oxidation end time (OET), length of oxidation Δt (OIT-OET), and rate of oxidation were assayed. The oxidation rate was calculated according to the following equation:

Oxidation Rate = (Y1 − Y2)/Δt(1)

where: Y1—heat flow at OIT point [W/g], Y2—heat flow at OET [W/g], Δt—length of oxidation.

### 4.6. Chemical Determination of Oxidative Stability

Measurements of p-anisidine value (pAV) for oil samples, as a measure of the level of secondary oxidative products, were carried out according to the ISO standard [34]. Spectrophotometric measurements were taken with a quartz cuvette with the 10 mm optical path length. A sample of 3 ± 0.001 g grams was weighed for measurement. The values obtained were calculated by means of the following equation:

pAV = (25 [1.2 (A1 − A2 − Ao)])/m(2)

where Ao is the absorbance of the non-reacting sample, A1 is the absorbance of the reacting sample, A2 is an absorbance of the blank sample and m is the mass of the sample [g]. 

Peroxide value was determined by following the ISO 3960 standard [35]. A sample of 5 ± 0.001 g grams was weighed for measurement. 

Calculations were performed using the following equation:

PV = ((V − Vo) × C_thio_ × F × 1000)/m (3)

where PV is peroxide value [meq O_2_/kg], V—volume of titrant in test portion [ml], V0—volume of titrant in blank [ml], C_thio_—molar concentration of the sodium thiosulfate solution in mol/l, F—exact concentration of the 0.01 N thiosulfate solution, m—weighed portion of test substance [g].

In accordance with the ISO 3960 standard [35], the total oxidation value (TOTOX) parameter was calculated, based on the pAV and PV values, by means of the following formula: 

TOTOX = pAV + 2PV, expressing the overall rate of oil oxidation.

Acid value (AV), as an indicator of the degree of hydrolytic changes, was measured in five oils according to the AOCS official method [36]. A sample of 10 ± 0.001 g was weighed for measurement. The resulting values were calculated using the following equation:

AV = ((A − B) × M × 56.1)/W(4)

where: AV—acid value [mg KOH/g of test portion], A—volume of standard alkali used in the titration [ml], B—volume of standard alkali used in titrating the blank [ml], M—molarity of standard alkali, W—mass of test portion [g]. 

### 4.7. Statistical Analysis of Results

The results were presented in the form of a mean and standard deviation. The first stage of the statistical analysis consisted of verifying ANOVA assumptions (variance homogeneity using the Hartley–Cochran–Bartlett test and data normality). If the assumptions were respected, one-way analysis of variance (ANOVA) was used and Tukey’s test was applied to create statistically homogeneous groups. In turn, when one assumption was not confirmed, non-parametric tests were used, i.e., ANOVA and the Kruskal–Wallis rank test. Pearson’s linear correlation coefficient was used to assess the significance of the relationship between selected variables. Additionally, principal component analysis (PCA) was used to assess the linear relationships between multiple variables. This analysis is an unsupervised pattern recognition method that is used for exploring raw data. This technique is also used to reduce the dimensionality of data sets. These methods define unique variances (principal components) using linear combinations of the original numeric variables, and these PCs are orthogonal (not correlated). Statistical analysis of the recorded results was performed using Tibco Statistica 13.3 software (Tibco Software Inc., Tulsa, Oklahoma, USA) at a significance level of α = 0.05.

## 5. Conclusions

The DSC technique allowed differences in the shape of crystallization and melting profiles obtained by different scanning rates to be identified, and the oxidative stability of five different flaxseed oil cultivars at various thermal conditions to be measured. The crystallization process measured using the DSC technique was manifested as a single peak, which among the cultivars ranged between −55.35 and −54.59 °C for a cooling rate of 1 °C/min, while for the scanning rate of 2 °C/min, the temperature ranged from −60.24 to −59.1 °C. Analysis of the melting process at different scanning rates revealed that melting curves were more complex, and in the case of rates 1 and 2 °C/min, the cultivars differed in curve shape, while the profiles did not differ for a scanning rate of 5 °C/min. Considering the results obtained from the melting process, it can be concluded that the scanning rate had a significant influence on the behavior of the oil during melting. The lower scanning rate of 1 °C/min affected the different melting behaviors among various cultivars, depending on the small differences in composition (content of unsaturated and saturated fatty acids). In the case of a higher scanning rate of 5 °C/min, the curves for all cultivars were similar and this fact could be utilized in analytics for profiling in order to assess the authenticity of flaxseed oil. Comparing the differences among flaxseed cultivars, it was observed that lower melting and crystallization temperatures were noted for oils characterized by the highest ratio of unsaturated fatty acids. Highly significant, negative linear correlation coefficients were obtained for the relation between the crystallization and melting peak temperatures and the ratio of UFA/SFA. The investigation of oxidation stability by means of the DSC technique revealed that flaxseed oil is very susceptible to thermal oxidation. The mean value calculated from all cultivars of time needed to start oxidation (OIT) at 120 °C was 42 min, while at 140 °C it was 10 min, and for 140 °C the rate of oxidation was three times higher than for 120 °C. In the DSC non-isothermal mode, it was possible to measure at which temperature oxidation starts. For the heating rate of 2 °C/min, the mean value from all cultivars of onset temperature for oxidation was 145 °C, and for a rate of 5 °C/min it was 159 °C. Significant linear correlations were found between unsaturated fatty acid content (C18:2, C18:3 n-3) and DSC parameters of isothermally and non-isothermally determined stability of flaxseed oils (OIT, Ton). Using PCA, it was also established that there is a strong negative correlation between PV, TOTOX values and all DSC parameters of the thermo-oxidative stability of flaxseed oil.

## Figures and Tables

**Table 1:** Fatty acid composition expressed as a percentage of total fatty acid (%). Saturated fatty acid (SFA), monounsaturated fatty acid (MUFA), polyunsaturated fatty acids (PUFA) and a ratio of n-3 to n-6 and unsaturated to saturated fatty acids (UFA /SFA) of different cultivars of cold-pressed flaxseed oils.

Fatty Acid	Flaxseed Variety				
	FL BU	FL DL	FL SZA	FL SZB	FL NN
12:0	0.04 ^a^ ± 0.00	0.03 ^a^ ± 0.00	0.03 ^a^ ± 0.00	0.04 ^a^ ± 0.01	0.04 ^a^ ± 0.01
14:0	0.02 ^a^ ± 0.00	0.02 ^a^ ± 0.01	0.02 ^a^ ± 0.01	0.02 ^a^ ± 0.00	0.02 ^a^ ± 0.00
16:0	4.86 ^a^ ± 0.03	4.96 ^ab^ ± 0.04	4.86 ^a^ ± 0.04	5.15 ^c^ ± 0.01	5.03 ^bc^ ± 0.05
16:1	0.05 ^a^ ± 0.01	0.06 ^a^ ± 0.00	0.06 ^a^ ± 0.00	0.06 ^a^ ± 0.01	0.09 ^a^ ± 0.02
17:0	0.07 ^a^ ± 0.00	0.05 ^a^ ± 0.00	0.06 ^a^ ± 0.01	0.07 ^a^ ± 0.01	0.08 ^a^ ± 0.04
17:1	0.04 ^a^ ± 0.01	0.04 ^a^ ± 0.01	0.04 ^a^ ± 0.00	0.04 ^a^ ± 0.00	0.04 ^a^ ± 0.01
18:0	3.00 ^a^ ± 0.01	4.01 ^b^ ± 0.01	3.99 ^b^ ± 0.04	4.30 ^c^ ± 0.02	4.54 ^d^ ± 0.02
18:1	14.64 ^a^ ± 0.01	16.64 ^c^ ± 0.01	16.13 ^b^ ± 0.03	18.46 ^d^ ± 0.06	18.71 ^e^ ± 0.00
18:2	16.56 ^d^ ± 0.03	14.77 ^c^ ± 0.07	11.14 ^a^ ± 0.14	11.88 ^b^ ± 0.01	14.92 ^c^ ± 0.08
18:3 n-6	0.21 ^a^ ± 0.03	0.20 ^a^ ± 0.03	0.18 ^a^ ± 0.00	0.17 ^a^ ± 0.02	0.20 ^a^ ± 0.01
18:3 n-3	59.96 ^d^ ± 0.05	58.77 ^b^ ± 0.05	63.11 ^e^ ± 0.16	59.39 ^c^ ± 0.06	55.91 ^a^ ± 0.15
20:0	0.15 ^a^ ± 0.05	0.15 ^a^ ± 0.034	0.14 ^a^ ± 0.01	0.15 ^a^ ± 0.01	0.14 ^a^ ± 0.01
20:1	0.14 ^a^ ± 0.01	0.10 ^a^ ± 0.03	0.09 ^a^ ± 0.01	0.12 ^a^ ± 0.01	0.13 ^a^ ± 0.04
20:3	0.06 ^a^ ± 0.00	0.05 ^a^ ± 0.01	0.03 ^a^ ± 0.04	0.02 ^a^ ± 0.03	0.02 ^a^ ± 0.03
22:0	0.13 ^a^ ± 0.01	0.12 ^a^ ± 0.01	0.12 ^a^ ± 0.01	0.12 ^a^ ± 0.00	0.14 ^a^ ± 0.01
24:0	0.08 ^a^ ± 0.01	0.08 ^a^ ± 0.00	0.05 ^a^ ± 0.06	0.05 ^a^ ± 0.06	0.05 ^a^ ± 0.06
ΣSFA	8.34	9.40	9.25	9.88	10.01
ΣMUFA	14.87	16.84	16.32	18.68	18.96
ΣPUFA	76.79	73.78	74.45	71.45	71.04
n-3/n-6	3.6	3.9	5.6	4.9	3.7
UFA/SFA	11.0	9.7	9.8	9.1	9.0

^abcde^—values are mean ± standard deviations of three measurements (*n* = 3), different superscript letters within rows indicate significant differences (*p* < 0.05). ΣSFA—total of saturated fatty acid. ΣMUFA—total of monounsaturated fatty acid. ΣPUFA—total of polyunsaturated fatty acid. UFA/SFA—ratio of total of unsaturated fatty acid to total of saturated fatty acid. FL BU (Bukoz Flaxseed cultivar), FL DL (Dolguniec Flaxseed cultivar), FL SZA, FL SZB (Szafir Flaxseed cultivar), FL NN (Unknown Flaxseed cultivar).

**Table 2:** CIE LAB L*, a*, b* values of cold-pressed flaxseed oils.

Seeds Variety	CIE LAB L*, a*, b* Values		
	L*	a*	b*
FL BU	87.86 ^e^ ± 0.06	8.99 ^e^ ± 0.07	136.58 ^e^ ± 0.06
FL DL	86.21 ^b^ ± 0.02	2.59 ^a^ ± 0.01	112.48 ^b^ ± 0.04
FL SZA	74.64 ^a^ ± 0.08	4.86 ^c^ ± 0.01	102.90 ^a^ ± 0.10
FL SZB	87.12 ^d^ ± 0.02	2.79 ^b^ ± 0.00	120.27 ^c^ ± 0.02
FL NN	86.64 ^c^ ± 0.00	5.09 ^d^ ± 0.01	128.31 ^d^ ± 0.03

^abcde^—values are mean ± standard deviations of three (*n* = 3) measurements, different superscript letters within rows indicate significant differences (*p* < 0.05). FL BU (Bukoz Flaxseed cultivar), FL DL (Dolguniec Flaxseed cultivar), FL SZA, FL SZB (Szafir Flaxseed cultivar), FL NN (Unknown Flaxseed cultivar).

**Table 3:** Differential scanning calorimetry (DSC) thermodynamic parameters of crystallization process of different cultivars of cold-pressed flaxseed oils obtained by different scanning rates.

Seeds Variety	Scanning Rate 1 °C/min		Scanning Rate 2 °C /min	
	Enthalpy	Peak Temperature	Enthalpy	Peak Temperature
	Δ H_c_ [J/g]	T_c_ [°C]	Δ H_c_ [J/g]	T_c_ [°C]
FL BU	−34.82 ^a^ ± 0.73	−55.35 ^a^ ± 2.01	−27.34 ^a^ ± 1.06	−60.24 ^a^ ± 1.90
FL DL	−34.33 ^a^ ± 0.42	−54.71 ^a^ ± 0.16	−27.87 ^a^ ± 0.54	−59.15 ^a^ ± 0.08
FL SZA	−34.71 ^a^ ± 0.21	−55.17 ^a^ ± 0.11	−28.49 ^a^ ± 0.90	−59.68 ^a^ ± 0.08
FL SZB	−34.66 ^a^ ± 1.31	−54.59 ^a^ ± 0.12	−28.99 ^a^ ± 1.06	−59.43 ^a^ ± 0.03
FL NN	−32.09 ^a^ ± 5.29	−54.74 ^a^ ± 0.01	−28.45 ^a^ ± 1.22	−59.10 ^a^ ± 0.07

^a^ value is mean ± standard deviations of three (*n* = 3) measurements. The same superscript letters within columns indicate no significant differences (*p* < 0.05). FL BU (Bukoz Flaxseed cultivar), FL DL (Dolguniec Flaxseed cultivar), FL SZA, FL SZB (Szafir Flaxseed cultivar), FL NN (Unknown Flaxseed cultivar).

**Table 4:** DSC thermodynamic parameters of melting process of different cultivars of cold-pressed flaxseed oils obtained by a scanning rate of 1 °C/min.

Seeds Variety	Peak Temperature [°C]		Enthalpy [J/g]		
	Tm1	Tm 2	Δ Hm Peak 1	Δ Hm Peak 2	Δ Hm Total
FL BU	−35.69 ^b^ ± 1.71	−13.77 ^b^ ± 1.60	35.12 ^b^ ± 6.82	92.80 ^d^ ± 7.99	127.91 ^c^ ± 13.44
FL DL	−32.45 ^a^ ± 0.18	−12.42 ^ab^ ± 0.23	19.09 ^a^ ± 1.75	66.26 ^a^ ± 0.22	85.35 ^b^ ± 1.66
FL SZA	−33.26 ^ab^ ± 0.20	−12.44 ^ab^ ± 0.51	24.67 ^ab^ ± 6.38	71.46 ^a^ ± 6.48	71.46 ^b^ ± 4.90
FL SZB	−31.43 ^a^ ± 0.38	−10.87 ^a^ ± 1.10	26.17 ^ab^ ± 0.28	8.09 ^b^ ± 1.26	34.27 ^a^ ± 1.53
FL NN	−31.73 ^a^ ± 0.22	−10.26 ^a^ ± 0.24	19.60 ^a^ ± 3.04	23.85 ^c^ ± 1.63	43.45 ^a^ ± 4.67

^abc^ values are mean ± standard deviations of three measurements (*n* = 3), different superscript letters within columns indicate significant differences (*p* < 0.05). Tm1—peak temperature for peak 1, counting from the lower to the higher temperatures. FL BU (Bukoz Flaxseed cultivar), FL DL (Dolguniec Flaxseed cultivar), FL SZA, FL SZB (Szafir Flaxseed cultivar), FL NN (Unknown Flaxseed cultivar).

**Table 5:** DSC thermodynamic parameters of melting process of different cultivars of cold-pressed flaxseed oils obtained by a scanning rate of 2 °C/min.

Seeds Variety	Peak Temperature [°C]		Enthalpy [J/g]		
	Tm1	Tm2	Δ Hm1	Δ Hm2	Δ Hm Total
FL BU	−32.89 ^a^ ± 2.03	−11.08 ^a^ ± 1.98	41.96 ^c^ ± 2.58	2.39 ^b^ ± 1.15	44.35 ^bc^ ± 1.88
FL DL	−30.66 ^a^ ± 0.19	−8.72 ^a^ ± 0.47	47.96 ^ab^ ± 2.00	1.28 ^ab^ ± 0.27	49.23 ^a^ ± 1.75
FL SZA	−31.49 ^a^ ± 0.19	−9.21 ^a^ ± 0.46	50.09 ^b^ ± 1.84	0.75 ^ab^ ± 0.14	50.84 ^a^ ± 1.76
FL SZB	−30.36 ^a^ ± 0.05	−8.56 ^a^ ± 0.26	48.14 ^ab^ ± 2.86	0.69 ^a^ ± 0.14	48.83 ^ac^ ± 2.97
FL NN	−30.34 ^a^ ± 0.07	nd	43.12 ^ac^ ± 1.95	nd	43.12 ^b^ ± 1.95

^abc^ values are mean ± standard deviations of three measurements (*n* = 3), different superscript letters within columns indicate significant differences (*p* < 0.05). nd: not detected. Tm1—peak temperature for peak 1, counting from the lower to the higher temperatures. Δ Hm1—enthalpy for peak 1, counting from the lower to the higher temperatures. FL BU (Bukoz Flaxseed cultivar), FL DL (Dolguniec Flaxseed cultivar), FL SZA, FL SZB (Szafir Flaxseed cultivar), FL NN (Unknown Flaxseed cultivar).

**Table 6:** DSC thermodynamic parameters of melting process of different cultivars of cold-pressed flaxseed oils obtained by a scanning rate of 5 °C/min.

Seeds Variety	Peak Temperature [°C]	Enthalpy [J/g]
	Tm1	Δ Hm
FL BU	−31.88 ^a^ ± 0.42	60.36 ^a^ ± 0.79
FL DL	−30.15 ^a^ ± 0.46	65.12 ^a^ ± 0.39
FL SZA	−30.53 ^a^ ± 0.62	61.33 ^a^ ± 1.20
FL SZB	−29.45 ^a^ ± 0.11	63.32 ^a^ ± 1.75
FL NN	−29.48 ^a^ ± 0.62	63.39 ^a^ ± 0.42

^a^ value is mean ± standard deviations of three measurements (*n* = 3), the same superscript letters within columns indicate no significant differences (*p* < 0.05). nd: not detected. FL BU (Bukoz Flaxseed cultivar), FL DL (Dolguniec Flaxseed cultivar), FL SZA, FL SZB (Szafir Flaxseed cultivar), FL NN (Unknown Flaxseed cultivar).

**Table 7:** Pearson’s correlation coefficients between fatty acid composition and oxidative stability parameters measured by DSC.

	C16:0	C18:0	C18:1	C18:2	18:3 n-3
OIT (120 °C)	0.08	0.37	0.39	0.53	−0.82 *
OIT (140 °C)	−0.21	−0.16	−0.07	0.80 *	−0.63 *
T_on_ (2 °C/min)	−0.13	0.18	0.17	0.47	−0.57
T_on_ (5 °C/min)	0.40	0.59	0.66 *	0.27	−0.82 *

* marked correlations are significant at *p* < 0.05.

## Data Availability

The data presented in this study are available upon reasonable request.

## Sample Availability

Not available.
